# Meningococcal Disease in South Africa, 1999–2002

**DOI:** 10.3201/eid1302.051553

**Published:** 2007-02

**Authors:** Garry B. Coulson, Anne von Gottberg, Mignon du Plessis, Anthony M. Smith, Linda de Gouveia, Keith P. Klugman, for the Group for Enteric, Respiratory, Meningeal Disease Surveillance in South Africa, GERMS-SA

**Affiliations:** * National Institute for Communicable Diseases of the National Health Laboratory Service, Johannesburg, South Africa; † Emory University, Atlanta, Georgia, USA

**Keywords:** *Neisseria meningitidis*, serogroup, meningococcal disease, ST-complex, hypervirulent strains, MLST, PFGE, research

## Abstract

We describe the epidemiology of invasive meningococcal disease in South Africa from August 1999 through July 2002, as reported to a laboratory-based surveillance system. *Neisseria meningitidis* isolates were further characterized. In total, 854 cases of laboratory-confirmed disease were reported, with an annual incidence rate of 0.64/100,000 population. Incidence was highest in infants <1 year of age. Serogroup B caused 41% of cases; serogroup A, 23%; serogroup Y, 21%; serogroup C, 8%; and serogroup W135, 5%. Serogroup B was the predominant serogroup in Western Cape Province, and disease rates remained stable. Serogroup A was most prevalent in Gauteng Province and increased over the 3 years. On pulsed-field gel electrophoresis analysis, serogroup A strains showed clonality, and serogroup B demonstrated considerable diversity. Selected isolates of serogroup A belonged to sequence type (ST)-1 (subgroup I/II) complex, serogroup B to ST-32/electrophoretic type (ET)-5 complex, and serogroup W135 to ST-11/ET-37 complex.

## Abstract 2

Serogroups and strains differ by location, although hypervirulent strains were identified throughout the country.

Despite progress in our understanding of the epidemiology of meningococcal disease, infection with *Neisseria meningitidis* continues to be a serious public health concern worldwide. Although occurring predominantly as sporadic disease with seasonal variation in most parts of the world, the highest burden of meningococcal disease occurs in the “meningitis belt” of sub-Saharan Africa, where epidemics are observed regularly (*1*). Historically these epidemics were associated with serogroup A and, to a lesser extent, serogroup C. However, serogroup W135 has recently emerged as a cause of epidemic disease in Africa (*2*,*3*), after outbreaks in 2000 and 2001 in Saudi Arabia during the annual Hajj pilgrimage to Mecca (*4*–*6*). Epidemics of meningococcal disease have occurred in Africa outside the meningitis belt (*7*,*8*).

Meningococcal disease associated with epidemics in Africa is generally caused by a limited number of genetically defined clonal groups (*9*,*10*). The 3 serogroup A pandemic waves reaching the African meningitis belt were caused by clones of subgroup III (*11*,*12*), and the recent outbreaks of W135 in West Africa were caused by strains belonging to the ET-37 complex (*3*,*13*).

In South Africa, meningococcal disease (a clinically reportable condition since 1920) is endemic, with seasonal increases during the winter months (*14*,*15*). Incidence rates, as determined by clinical notifications to the Department of Health, have been steadily decreasing from ≈5–10/100,000 (1945–1975) to <2/100,000 (1992–1997) (*16*–*18*). Upsurges of disease with a periodicity of several years have been noted (*17*). During the late 1970s, the epidemiology changed from a preponderance of disease due to serogroup A in young adult black men on the gold mines in the Southern Transvaal (now Gauteng) Province, to mostly serogroup B disease affecting young mixed-race infants in Western Cape Province (*15*,*16*). Serogroup B has caused peaks in disease rates in Western Cape in 1979 (*9*,*19*–*21*) and again in 1988. Although predominant in Western Cape, serogroup B also caused cases in Johannesburg, Gauteng, during 1980–1982, where >60% of meningococcal disease in children was due to serogroup B (*22*). Increases in serogroup A disease in Gauteng were described in the 1980s and 1996 (*18*,*23*,*24*).

To better understand the recent epidemiology of invasive meningococcal disease in South Africa, we analyzed cases reported to a national laboratory-based surveillance system for a 3-year period, from August 1999 through July 2002. Isolates available from cases reported during this period were characterized further.

## Materials and Methods

### Case Definition

National laboratory-based surveillance for invasive disease caused by *N. meningitidis* is performed by the Respiratory and Meningeal Pathogens Research Unit (RMPRU) at the National Institute for Communicable Diseases (a branch of the National Health Laboratory Service) in Johannesburg, South Africa. Cases were defined as isolation of *N. meningitidis* from normally sterile body fluid specimens (blood, cerebrospinal fluid [CSF], or both) from patients in August 1999 through July 2002. Isolates were submitted voluntarily to RMPRU by ≈100 laboratories nationally. Laboratories were encouraged to submit case reports of laboratory-confirmed disease even if viable isolates were no longer available for submission. (Some isolates lost viability during transport to the central laboratory.) Annual audits were performed to ascertain missed cases, and these were included on the database (but were without viable isolates for further testing at the central laboratory). These audits identified 118 cases not reported, to reach a final total of 557 cases from the provinces/laboratories audited, which suggests that ≈70%–80% of laboratory-confirmed cases were reported to the surveillance system.

### Serogrouping

Serogroup was determined for 615 isolates by using latex slide agglutination with monoclonal antiserum to capsular polysaccharides A, B, C, X, Y, Z, and W135 (Murex Biotech Limited, Dartford, England, United Kingdom). Strains that did not react with these antisera were sent to the World Health Organization Collaborating Center for Reference and Research on Meningococci, Oslo, Norway, for serogrouping.

### Pulsed-Field Gel Electrophoresis (PFGE)

PFGE was performed on 573 viable isolates of serogroup A, B, C, W135, and Y meningococci by using a method adapted from Popovic et al. (*25*). PFGE restriction profiles were analyzed with the GelCompar version 4.1 software (Applied Maths, Kortrijk, Belgium). Dendrograms were created by using the unweighted pair group method with arithmetic averages. Analysis of the banding patterns was performed with the Dice coefficient and a position tolerance of 1.5% for the band migration distance. A PFGE cluster was defined as >3 isolates sharing >80% similarity on the dendrogram (*25*,*26*).

### Multi-Locus Sequence Typing (MLST)

MLST was performed on 46 isolates as described by Maiden et al. (*27*). We made use of the Neisseria MLST website (http://pubmlst.org/neisseria/) sited at the University of Oxford (*28*).

### Statistical Analysis

Incidence rates were calculated on the basis of the number of cases reported during the 12-month periods from August 1 through July 31 of the following year, divided by mid-year population estimates for years 2000, 2001, and 2002, respectively, obtained from the South African Health Information Systems Programme. The χ^2^ test for linear trend using EpiInfo 6 (version 6.04d; Centers for Disease Control and Prevention, Atlanta, Georgia, USA) was used to assess statistical significance of the changes during the 3-year period.

## Results

### Epidemiology of Laboratory-confirmed Meningococcal Disease

**Figure 1:**
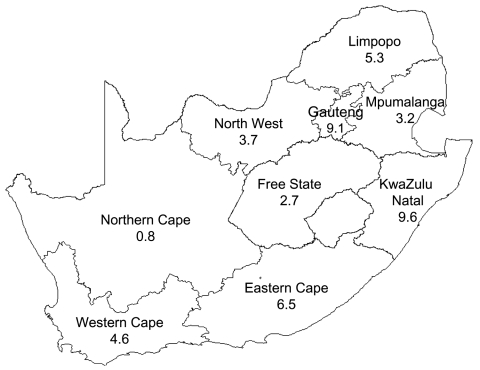
Map of South Africa with estimated provincial populations in 2002 (45.5 million [m] population). Values in boxes are in millions.

**Figure 2:**
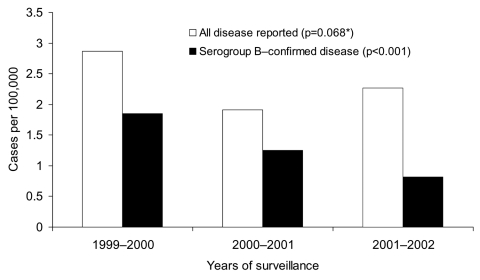
Incidence rates for all reported and serogroup B–confirmed meningococcal disease by year in Western Cape Province. *χ^2^ test for trend.

**Figure 3:**
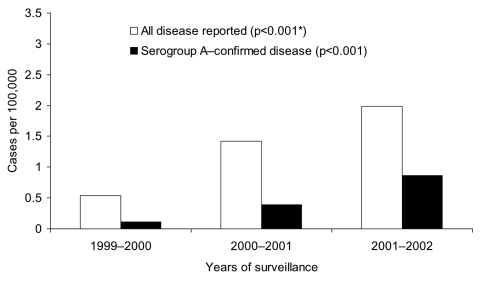
Incidence rates for all reported and serogroup A–confirmed meningococcal disease by year in Gauteng Province. * χ^2^ test for trend.

From August 1999 through July 2002, 854 cases of invasive meningococcal disease were reported; age was known for 756 (88%) patients. Most cases (645, 76%) were diagnosed from positive culture of CSF specimens (with or without positive cultures from blood specimens); the other 209 (24%) were positive on blood culture alone. The incidence rates of disease reported to the network increased from 0.52 per 100,000 persons in 1999–2000, to 0.62 in 2000–2001, and 0.77 in 2001–2002 (p<0.001). Western Cape Province was responsible for 37% of cases reported nationally, and Gauteng Province was responsible for 41% of cases reported nationally (Figure 1). In Western Cape Province, disease rates remained relatively stable; rates of reported disease were calculated as 2.87/100,000, 1.91/100,000, and 2.27/100,000 for each 12-month period, respectively (p = 0.068) (Figure 2). The incidence rates in Gauteng Province increased from 0.54/100,000 in the first year to 1.42/100,000 and 1.99/100,000 in the subsequent 2 years (p<0.001) (Figure 3). Seasonal variation was observed; the highest number of cases was reported in July to October (winter and spring) (data not shown). The highest age-specific incidence of meningococcal disease was seen in infants <1 year of age; the average incidence rate was 6.7/100,000. One hundred eighty-two (24%) of patients were infants <1 year of age, 116 (15%) were children 2–4 years of age, and 127 (17%) were young adults 15–24 years of age.

### Serogroup-specific Disease

Of the 854 cases of laboratory-confirmed meningococcal disease reported to the surveillance network, 615 (72%) had viable isolates available for serogrouping; 453 (74%) of these were isolated from CSF and 162 (26%) from blood culture alone. Serogrouping of the viable meningococcal isolates showed the following: serogroup B, 251 isolates (41%); A, 142 (23%); Y, 130 (21%); C, 50 (8%); W135, 31 (5%); X, 8 (1%); Z, 2 (<1%); and 29E, 1 (<1%) (Table 1).

**Table 1:** Provincial distribution of reported invasive meningococcal disease by serogroup and date of study, South Africa

Year	Serogroup	Province*									Total
		EC	FS	GA	KZ	LIM	MP	NC	NW	WC	N (%)†
Aug 1999–Jul 2000	A	–‡	–	9	1	–	–	–	1	4	15 (8)
	B	6	–	12	4	–	3	–	–	81	106 (56)
	C	2	2	2	–	–	–	–	–	14	20 (10)
	W135	–	–	3	3	–	–	–	–	4	10 (5)
	X	–	–	1	–	–	–	–	–	2	3 (2)
	Y	2	6	10	7	–	–	–	–	11	36 (19)
	No isolate available	2		9	10	4	2		2	10	39
	Total	12	8	46	25	4	5	–	3	126	229
Aug 2000–Jul 2001	A	–	–	34	2	–	5	–	3	–	44 (23)
	B	6	3	13	3	–	1	–	1	56	83 (43)
	C	3	–	3	–	–	1	–	1	4	12 (6)
	W135	–	–	7	–	–	–	–	–	1	8 (4)
	X	–	–	1	–	–	–	–	–	1	2 (1)
	Y	2	14	13	1	2	3	1	1	5	42 (22)
	Z	–	–	2	–	–	–	–	–	–	2 (1)
	No isolate available	3		51	2	6	1			19	82
	Total	14	17	124	8	8	11	1	6	86	275
Aug 2001–Jul 2002	A	–	–	78	1	–	1	–	2	1	83 (36)
	B	3	4	15	–	–	–	1	1	38	62 (27)
	C	1	–	6	–	–	–	1	–	10	18 (8)
	29E	–	–	–	–	–	–	–	–	1	1 (<1)
	W135	1	–	9	–	–	–	–	1	2	13 (6)
	X	–	–	2	–	–	–	–	–	1	3 (1)
	Y	3	14	20	4	–	1	2	1	7	52 (22)
	No isolate available	3	2	51	5		10	1	1	45	118
	Total	11	20	181	10	–	12	5	6	105	350

*EC, Eastern Cape; FS, Free State; GA, Gauteng; KZ, KwaZulu Natal; LIM, Limpopo; MP, Mpumalanga; NC, Northern Cape; NW, North West; WC, Western Cape. †Percentages denote the percentage of that particular serogroup over the total number of serogrouped isolates for that year. ‡No cases reported.

Seventy percent (175/251) of serogroup B disease was reported from Western Cape Province, where the number of cases decreased progressively from 81 in the first year (1999–2000) to 38 (in the third year, 2001–2002) (Table 1) and incidence decreased from 1.85/100,000 to 0.82, respectively (p<0.001) (Figure 2 ). Eighty-five percent (121/142) of serogroup A disease came from Gauteng Province, and the annual number of cases increased from 9 to 78 during the study period (Table 1). The incidence rate increased from 0.11/100,000 in the first year to 0.86 in the third year (p<0.001) (Figure 3). The proportion of disease caused by serogroups C, W135, and Y remained stable during the 3-year period (Table 1). Serogroup W135 was most prevalent in Gauteng Province (19 [61%] of the 31 cases occurred there), and serogroup C was most prevalent in Western Cape Province (28 [56%] of 50).

**Figure 4:**
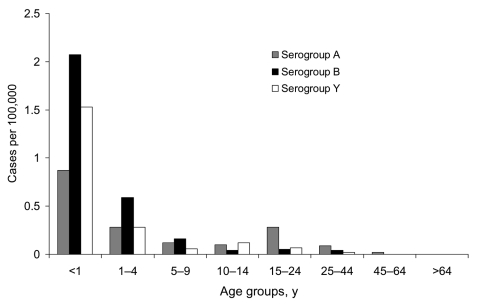
Annual age-specific incidence rates for confirmed serogroup A, B, and Y meningococcal disease in South Africa, as reported from August 2001 through July 2002.

The age-specific proportion of disease in patients with known age varied for serogroups. The highest proportion of serogroup A (38 [33%] of 114) and C (10 [20%] of 50) disease occurred in the 15- to 24-year age group; the highest proportion of disease caused by serogroup B (70 [29%] of 238) and Y (42 [38%] of 112) was in infants <1 year of age. Serogroup W135 was found in equal proportion in the <1-year age group (6 [23%] of 26) and 15–24 age group (7 [27%] of 26). Incidence rates for the most common serogroups (A, B, and Y) for the last year of surveillance showed the highest rates of disease in children <1 year of age (Figure 4). Serogroup A had the lowest rates of disease for infants of the 3 serogroups and also had a second small peak for young adults. These trends were similar in the previous 2 years.

Of 615 *N. meningitidis* isolates serogrouped, 573 (93%) isolates were characterized by PFGE. Forty-six of these isolates were selected for MLST (Table 2).

**Table 2:** Genotypic data of *Neisseria meningitidis* isolates causing invasive disease as reported in South Africa, August 1999 through July 2002*

Serogroup	PFGE clusters	No. of isolates				MLST results		
		1999/2000, n	2000/2001, n	2001/2002, n	Total, N (%)	ST	ST complex	n
A	Total	13	38	72	123			
	Cluster A-1	5	34	70	109 (89)	1	ST-1/subgroup I/II	12
	Small clusters/single isolates	8	4	2	14 (11)	7	ST-5/subgroup III complex	2
						254	ST-254 complex	1
						175	None	1
B	Total	107	77	58	242			
	Cluster B-1	38	28	25	91 (38)	33	ST-32/ET-5 complex	4
						4239	ST-32/ET-5 complex	1
	Cluster B-2	12	7	10	29 (12)	154	ST-41/44/lineage III	2
						4242	ST-41/44/lineage III	1
	Cluster B-3	11	9	3	23 (9.5)			
	Cluster B-4	7	4	6	17 (7)	35	ST-35 complex	1
	Cluster B-5	3	6	5	14 (6)			
	Small clusters/single isolates	36	23	9	68 (28)			
C	Total	20	12	17	49			
	Cluster C-1	5	5	5	15 (31)	11	ST-11/ET-37 complex	2
	Cluster C-2	3	2	4	9 (18)	865	None	2
	Cluster C-3	4	1	4	9 (18)	33	ST-32/ET-5 complex	2
	Small clusters/single isolates	8	4	4	16 (33)			
W135	Total	9	9	13	31			
	Cluster W-1	5	7	10	23 (74)	11	ST-11/ET-37 complex	4
	Cluster W-2	2	1	0	3 (10)	4241	ST-4241/ST-22 complex	1
	Small clusters/single isolates	2	1	3	5 (16)			
Y	Total	40	38	50	128			
	Cluster Y-1	28	25	39	92 (72)	175	None	6
	Cluster Y-2	5	8	6	19 (15)	23	ST-23 complex/cluster A3	2
						4245	ST-23 complex/cluster A3	1
	Small clusters/single isolates	7	5	5	17 (13)	175	None	1

*PFGE, pulsed-field gel electrophoresis; MLST, multilocus sequence typing; ST, sequence type.

### Molecular Epidemiology

#### Serogroup A

**Figure 5:**
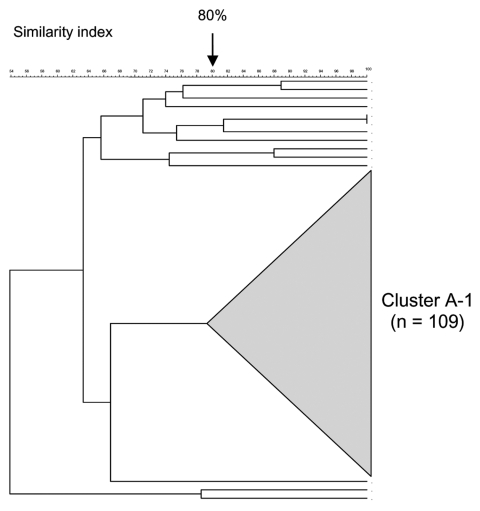
Pulsed-field gel electrophoresis dendrogram indicating the genetic relationship among serogroup A meningococcal isolates in South Africa, August 1999–July 2002.

PFGE analysis of 123 serogroup A isolates showed a highly clonal population structure with a large cluster (cluster A-1) representing 89% (109/123) (Figure 5, Table 2). The proportion of serogroup A meningococcal disease associated with strains of cluster A-1 increased from 38% (5/13) in 1999–2000 to 97% (70/72) in 2001–2002 (p<0.001). Most isolates from this cluster (101/109, 93%) originated from Gauteng Province and increased from 56% (5/9) in the first year, to 97% (30/31 and 66/68, respectively) in the second and third years (p<0.001). MLST analysis of 12 isolates from cluster A-1 showed identical allelic profiles belonging to sequence type (ST)-1, the prototype ST for the ST-1 (subgroup I/II) complex (Table 2). MLST analysis of 4 isolates outside of cluster A-1 yielded strains belonging to ST-7 (n = 2), ST-254 (n = 1), and ST-175 (n = 1 (Table 2).

#### Serogroup B

**Figure 6:**
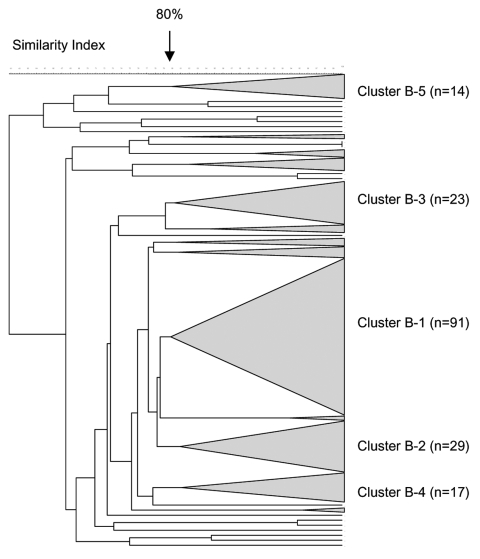
Pulsed-field gel electrophoresis dendrogram indicating the genetic relationship among serogroup B meningococcal isolates in South Africa, August 1999–July 2002.

In total, 242 serogroup B *N. meningitidis* isolates were analyzed by PFGE. Five distinct clusters were observed, with a predominant cluster (cluster B-1) consisting of 38% (91/242) of the isolates (Figure 6). The proportion of isolates within this cluster was 36%, 36%, and 43% for each 12-month period, respectively (p = 0.369). Eighty-two percent (75/91) of the isolates from this cluster were from the Western Cape, and the proportion of these strains in this province remained stable over time. Five isolates from this cluster were selected for MLST analysis. Four isolates were ST-33 (Table 2). One isolate had a novel allele at the *fumC* locus (*28*); a new ST (ST-4239, still part of ST-32 complex) was assigned to this isolate.

The second largest cluster (cluster B-2) comprised 12% (29/242) of the total number of isolates characterized. Three isolates belonged to ST-41/44 lineage III, 2 of which were ST-154. The third isolate had a novel allele at the *abcZ* locus and was assigned ST-4242.

Clusters B-3, B-4, and B-5 comprised 9.5% (23/242), 7% (17), and 6% (14) of all serogroup B isolates, respectively. The remaining isolates were clustered into small groups or were unrelated.

#### Serogroup C

PFGE of the 49 serogroup C meningococcal isolates showed 3 main clusters (clusters C-1, C-2, and C-3) (Table 2). Cluster C-1 comprised 31% (15/49) of the total number of serogroup C isolates and showed no particular concentration by province. Two isolates from this cluster were ST-11 (Table 2).

Isolates belonging to clusters C-2 and C-3 each made up 18% (9/49) of the total number of isolates characterized (Table 2). Isolates from both clusters came exclusively from Western Cape (18/18 isolates). MLST of 2 strains from each cluster identified ST-865 in cluster C-2 (an ST not associated with any broader ST complex), and ST-33 in cluster C-3 (Table 2). The remaining isolates all showed unrelated PFGE patterns.

#### Serogroup W135

Of the 31 serogroup W135 meningococci isolates analyzed by PFGE, a distinct cluster (cluster W-1) of isolates comprising 23 (74%) of 31 isolates was found (Table 2). Seventeen (74%) of cluster W-1 isolates came from Gauteng Province. MLST analysis of 4 isolates from cluster W-1 (2 isolates from Gauteng and 1 each from Western Cape and KwaZulu Natal Provinces) showed they were ST-11, the founder sequence type of the ST-11/electrophoretic type (ET)-37 complex. Cluster W-2 comprised 3 isolates from 3 provinces, and MLST of 1 of the isolates showed that it belonged to ST-4241 (ST-22 complex). The remaining isolates were unrelated.

#### Serogroup Y

PFGE analysis of the 128 serogroup Y meningococcal isolates showed 2 clusters (clusters Y-1 and Y-2; Table 2). The predominant cluster (cluster Y-1) consisted of 92 (72%) isolates. Twenty-five (27%) isolates from cluster Y-1 came from Free State Province, 27 (29%) from Gauteng Province, and 15 (16%) from Western Cape Province. MLST of 6 isolates from this cluster showed that they were ST-175 (Table 2).

A second cluster, cluster Y-2, comprised 15% (19/128) of isolates (Table 2). Fifty-three percent (10/19) of these isolates were from Gauteng Province. MLST analysis of 3 isolates showed 2 STs, 1 identified as ST-23 (2 isolates), with the third possessing a novel allele at the *abcZ* locus (assigned new ST-4245). The remaining isolates (17/128, 13%) demonstrated groups of 2, 3, or 5 isolates; and 4 unrelated isolates. MLST analysis of 1 of these isolates showed that it belonged to ST-175.

## Discussion

The endemic nature and low incidence rates of meningococcal disease in the study period confirm an epidemiology related more closely to industrialized countries (*29*,*30*) than to countries of the African meningitis belt. Rates of national disease, as calculated by clinical notifications, ranged between 1 and 2/100,000 from 1992 to 1997 (*18*) and are similar to those calculated in our study. Although laboratory-based surveillance in South Africa clearly underestimates the impact of disease, audits indicate that more than two thirds of laboratory-confirmed disease were reported, and we believe our data are representative enough to reflect general trends of disease.

Overall, the age group at greatest risk for disease was children <1 year of age, although there were some differences by serogroup. Serogroup B has been previously described to occur predominantly in infants (*15*,*22*,*30*); serogroup A disease also causes disease in adults (*15*,*22*,*24*). Serogroup Y disease occurring in older patients has been documented (*30*), but this was not observed in our study.

The high proportion of laboratory-confirmed cases from Gauteng and Western Cape Provinces could reflect better reporting by laboratories in these areas. These 2 provinces also had the most clinical notifications, which would be less reliant on laboratory facilities, to the Department of Health since the 1970s (*18*). Other parts of South Africa were noted to have much lower rates of disease (*15*). Although access to medical care may influence rates by province, the fulminant and distinctive clinical manifestations of meningococcal disease allow for adequate clinical reporting from health facilities. True environmental, socioeconomic, or host-related factors may be resulting in higher disease rates in these provinces. Climate varies between areas in the country: Western Cape has a Mediterranean climate with wet winters and hot, dry summers; Gauteng lies on a plateau and has a temperate climate with summer rainfall; and KwaZulu Natal has a predominantly subtropical climate (*31*).

The incidence rate of reported meningococcal disease increased from 1999 to 2002, and serogroup A, most prevalent in Gauteng Province, was the only serogroup of viable isolates to increase significantly. Cyclical changes in meningococcal disease occurring every 8 to 10 years have been noted in this province (*18*). Case ascertainment of prospectively reported cases may have increased as the surveillance became more established and as audits highlighted nonreporting from certain laboratories that were subsequently included in the surveillance. Serogroup A meningococci are associated with most outbreaks throughout the African meningitis belt (*1*). No discrete outbreaks were identified associated with serogroup A disease during the study period; however, unrecognized clusters may have occurred.

The increase in the number of cases of serogroup A reported from Gauteng Province was associated specifically with strains belonging to a distinct cluster identified by PFGE. Selected isolates from this cluster were confirmed as belonging to ST-1 (subgroup I/II) complex. These strains have caused epidemics worldwide (*11*,*32*,*33*). In South Africa, subgroup I strains were first identified in 1968 (1 isolate) (*11*,*32*) and from 1976 through 1983 (41 isolates) (*32*). In 1996, 49.5% (55/111) of isolates analyzed from an outbreak in South Africa were identified as serogroup A belonging to subgroup I; 13.5% (15/111) belonged to subgroup III (*34*). MLST analysis of 1 of these subgroup III isolates showed it was ST-5 (data not shown). Recently, in the meningitis belt, ST-5 (predominant in 1988–2001) has been replaced by ST-7 in 2002, and no ST-1 strains were identified (*35*). In our study, 2 isolates analyzed by MLST were confirmed as ST-7, which suggests that the third pandemic wave from People’s Republic of China may have reached South Africa (*12*,*35*). The predominant serogroup A strain causing disease in South Africa, however, was not the same strain as that in the meningitis belt.

The high proportion of sporadic serogroup B disease in the Western Cape has been well described since the late 1970s (*15*,*19*,*36*). Serogroup B is rarely reported from other countries in Africa, and our data reflect an epidemiology for this serogroup more consistent with industrialized countries (*9*,*37*). The proportion of serogroup B meningococcal disease nationally decreased significantly, mostly due to a decrease in the number of viable serogroup B isolates identified from the Western Cape. This province had no change in total reported disease rates. By PFGE this serogroup showed substantial diversity, a characteristic typical of sporadic serogroup B disease worldwide (*30*,*38*) and previously documented in the Western Cape (*39*). Complexes ST-32/ET-5 and ST-41/44/lineage III have been associated with outbreaks worldwide (*9*,*37*). These strains have been causing disease in the Western Cape since the late 1970s (*9*,*21*,*39*).

Serogroup Y accounts for approximately one third of all invasive meningococcal disease in the United States (*30*), but it has been rare in the African meningitis belt (*1*,*35*). A serogroup Y isolate with ST-175 has been previously described from The Gambia in 1988 (www.pubmlst.org/neisseria), and recently ST-23 and ST-2880 have been identified in the meningitis belt (*35*). Our data thus represent the first evidence of a major role for serogroup Y disease in Africa.

Serogroup C disease associated with sporadic disease and occasional outbreaks occurs in both industrialized and developing nations (*1*,*30*). Complexes ST-11/ET-37 and ST-32/ET-5 are hypervirulent meningococci reported worldwide (*9*,*37*). In South Africa, a community-based outbreak caused by strains of the ST-11/ET-37 complex was observed in 2003 (*40*). ST-865 strains have been reported to cause disease in Taiwan, Spain, and the United States (www.pubmlst.org/neisseria), but these were associated with nongroupable and non–serogroup C isolates. To our knowledge, we document the first serogroup C strain of ST-865.

Serogroup W135, associated with little disease worldwide (*1*,*13*), represented a small proportion of disease in our surveillance. Complex ST-11/ET-37 was responsible for outbreaks in 2000 and 2001 associated with the annual Hajj pilgrimage (*4*,*6*,*13*) and for outbreaks in Burkina Faso in 2001 (*2*). PFGE comparison of isolates from a predominant cluster in South Africa with an isolate from the Hajj outbreak showed that they were related (data not shown). Strains of ET-37 had been in South Africa in 1986 and 1990 (*13*), and this clone may have been reintroduced during the Hajj outbreak.

In conclusion, we identified sporadic and seasonal meningococcal disease in South Africa during the study period, caused by an increasing number of cases due to a clone of serogroup A in Gauteng Province. Diverse strains of serogroup B were responsible for stable prevalence of disease in Western Cape Province. Nationally, 21% of meningococcal disease was due to serogroup Y. Continued surveillance will provide valuable information for the development of public health strategies to minimize the risk for outbreaks in South Africa and neighboring countries.
